# Targeted nanopore long-read sequencing panel for the molecular diagnosis of intronic expansion in familial adult myoclonic epilepsy

**DOI:** 10.1186/s12920-025-02247-9

**Published:** 2025-11-11

**Authors:** Haruka Urabe, Takashi Nakajima, Satomi Mitsuhashi, Kentaro Ohta, Hidehiko Fujinaka, Kiyoe Goto, Aki Sato

**Affiliations:** 1 Department of Neurology, NHO Niigata National Hospital, 3-52 Akasaka-cho, Kashiwazaki, 945-8585 Japan; 2 Department of Neurology, St. Marianna University School of Medicine, 2-16-1 Sugao, Miyamae-ku, Kawasaki, 216-8511 Japan; 3 Department of Clinical Genetics, NHO Niigata National Hospital, 3-52 Akasaka- cho, Kashiwazaki, 945-8585 Japan

**Keywords:** Long-read sequencing, Gene panel, Familial adult myoclonic epilepsy, Benign adult-onset familial myoclonic epilepsy, Repeat diseases

## Abstract

**Background:**

Familial adult myoclonic epilepsy (FAME), an autosomal dominant disorder, is characterized by cortical myoclonus and occasional generalized tonic–clonic seizures. To date, intronic pentanucleotide repeat expansions in at least seven genes, including *SAMD12*, *TNRC6A*, *YEATS2*, *MARCHF6*, *STARD7*, *RAPGEF2*, and *RAI1*, have been reported as causative. Detecting these repeat expansions using conventional sequencing techniques (Sanger or short-read next-generation sequencing) is not feasible as they cannot reliably span or characterize long repetitive elements. Although genetic testing has been performed in some research laboratories, comprehensive long read–based panel is unavailable for clinical application. To address this gap, we developed a targeted long-read sequencing panel and applied it in a clinical diagnostic context for the first time.

**Methods:**

We designed a custom long-read sequencing panel targeting all seven known FAME-associated repeat loci using Oxford Nanopore Cas9-enrichment technology and applied it to a 47-year-old woman with familial cortical myoclonic tremor, clinically suspected to have FAME.

**Results:**

The panel functioned as intended, providing robust on-target coverage across all loci, facilitating confident interrogation of each repeat region. At the *SAMD12* locus, strand-aware histograms and read-level inspection demonstrated a clear pathogenic expansion, encompassing mixed TTTTA/TTTCA motifs with detectable TTTGA interruptions, consistent with FAME1. Using the crude allele prediction option of tandem-genotypes, the expanded allele contained approximately 689 additional repeats relative to the reference genome. The other six loci showed no pathogenic expansions.

**Conclusions:**

This targeted long-read panel enabled the first clinical molecular diagnosis of FAME using a comprehensive assay, yielding allele-resolved characterization of the pathogenic repeat and its motif composition. With further validation, this approach may serve as a clinically practical tool for reliable detection of FAME1 and for broader screening of other FAME subtypes, potentially reducing reliance on prolonged clinical observation or specialized electrophysiological testing.

**Supplementary Information:**

The online version contains supplementary material available at 10.1186/s12920-025-02247-9.

## Background

Familial adult myoclonic epilepsy (FAME), historically termed benign adult-onset familial myoclonic epilepsy (BAFME), is characterized by cortical myoclonic tremor and occasional generalized tonic–clonic seizures [1, 2]. Cortical tremor most often develops after the second decade of life; however, onset may occur much earlier in some cases owing to anticipation. Current classifications designate BAFME as FAME1, a subtype of FAME. Although FAME is generally regarded as a self-limited epileptic syndrome, some patients develop generalized tonic–clonic seizures later in their lives [3, 4].

Clinical features and electrophysiological findings, including giant somatosensory evoked potentials and cortical reflex myoclonus, mainly serve as the basis for diagnosis. Recent studies have identified intronic pentanucleotide repeat expansions as the genetic cause of FAME. Notably, the first causative repeat expansion in *SAMD12* (FAME1) remained undiscovered until the advent of long-read sequencing, as conventional methods, including Sanger sequencing or short-read next-generation sequencing (NGS), did not detect the expansion. Subsequent long-read studies established pathogenic expansions in additional FAME genes, including *SAMD12* [5], *STARD7* [6], *MARCHF6* [7], *YEATS2* [8], *TNRC6A* [5], *RAPGEF2* [5], and *RAI1* [9]. Each gene is associated with a distinct subtype of the disease, designated as FAME1–FAME8. However, FAME5 has not yet been linked to a causative gene. These repeat expansions are located in noncoding regions and are typically composed of motifs, including TTTTA and TTTCA.

Conventional sequencing methods, including Sanger sequencing or short-read NGS, fundamentally cannot resolve these pathogenic expansions because PCR amplification introduces severe bias, and short reads cannot span or map across long repetitive elements. Owing to these limitations, attempts have been made to utilize short read–based tools, including ExpansionHunter [10] or ExpansionHunter Denovo [11]. Although these tools may occasionally suggest the presence of an expansion, they cannot reliably size or fully characterize these loci, particularly in the case of large or interrupted repeats [12]. Conversely, long-read sequencing offers direct allele-resolved evidence of the pathogenic repeat and its motif composition, facilitating an unambiguous molecular diagnosis [13].

Currently, few research laboratories perform genetic testing for FAME, and no comprehensive long read–based panel has been developed for simultaneously assessing all known FAME-associated loci. Therefore, despite the strong clinical suspicion of FAME, genetic confirmation has remained difficult in practice, as a comprehensive diagnostic panel is unavailable. To address this gap, we designed a targeted long-read sequencing panel covering all seven known FAME-associated loci. The present case represents the first clinical application of this custom panel, undertaken because conventional methods were not expected to yield a definitive diagnosis. This strategy facilitated an unambiguous identification of the pathogenic repeat expansion.

We here developed and applied a custom long-read sequencing panel targeting all seven known FAME-associated loci to a patient with suspected FAME. This approach aimed to develop a comprehensive and reliable method for the molecular diagnosis of FAME. This panel could potentially facilitate a definitive diagnosis of FAME without necessitating extended clinical observation or detailed electrophysiological assessment.

## Methods

### FAME repeat panel for Cas9-mediated nanopore sequencing

Genomic DNA was extracted from the patient’s peripheral lymphocytes using the QIAamp DNA Blood kit (Qiagen, Venlo, The Netherlands), and then purified and concentrated with AMPure XP magnetic beads (Beckman Coulter, Brea, CA, USA). According to the manufacturer’s protocol, 10 µg of input DNA is recommended for Cas9 enrichment. We used 10 µg of input DNA in this study. Guide RNAs (crRNAs) targeting the seven known FAME-associated repeat loci were designed using the Alt-R CRISPR-Cas9 guide RNA design tool (Integrated DNA Technologies, Coralville, IA, USA). To generate the FAME repeat-target panel, the crRNAs were pooled in equimolar concentrations (Table 1). Cas9-mediated targeted enrichment and nanopore library preparation were performed using the Cas9 enrichment kit (SQK-CS9109, Oxford Nanopore Technologies, Oxford Science Park, Oxford, U.K.) according to the manufacturer’s instructions and previously described protocols [14]. Sequencing was conducted on the GridION platform (Oxford Nanopore Technologies) using a MinION flow cell per sample. Long-read sequencing was performed on Oxford Nanopore R9.4.1 (FLO-PRO002, Oxford Nanopore Technologies) flow cells using the Cas9 enrichment kit. In this study, raw signal data were collected using MinKNOW version 23.11.7 (Oxford Nanopore Technologies). Basecalling was performed using Dorado version 7.2.13 using the High-Accuracy model (450-bps mode).

**Table 1:** Summary of FAME-associated pentanucleotide repeat loci targeted in this study

Disease	Inheritance	Locus	Repeat unit	Gene	Function
FAME1 (BAFME)	Autosomal dominant	chr8:118366815–118366918	AAATA	*SAMD12*	Intron
FAME2	Autosomal dominant	chr2:96197066–96197124	AAAAT	*STARD7*	Intron
FAME3	Autosomal dominant	chr5:10356339–10356411	AAAAT	*MARCHF6*	Intron
FAME4	Autosomal dominant	chr3:183712187–183712226	TTTTA	*YEATS2*	Intron
FAME6	Autosomal dominant	chr16:24613438–24613532	AAAAT	*TNRC6A*	Intron
FAME7	Autosomal dominant	chr4:159342526–159342618	AAAAT	*RAPGEF2*	Intron
FAME8	Autosomal dominant	chr17:17808359–17808460	TTTTA	*RAI1*	Intron

### Data analysis for long-read sequencing

The reference genome used was hg38 (UCSC); subsequently, the sequencing error rates were calculated using LAST-TRAIN [15]. The command used for this calculation is described at https://gitlab.com/mcfrith/last/-/blob/main/doc/last-cookbook.rst or elsewhere. Repeat expansion was analyzed using the tandem-genotypes tool, available at https://github.com/mcfrith/tandem-genotypes, following the method described by Mitsuhashi, Frith et al. [16]. The repeat sequence of the expanded allele was visualized as a waterfall plot using RepeatAnalysisTools, available at https://github.com/PacificBiosciences/apps-scripts/tree/master/RepeatAnalysisTools.

On- and off-target scores for each guide RNA were obtained from the IDT Alt-R CRISPR-Cas9 guide RNA design tool (Integrated DNA Technologies, https://www.idtdna.com). According to the company’s website, the on-target score represents the predicted cleavage efficiency of the guide RNA at the intended genomic locus, whereas the off-target score indicates the predicted specificity, i.e., the likelihood of avoiding cleavage at unintended genomic sites. Both scores are reported on a 0–100 scale, with higher scores indicating greater predicted efficiency (on-target) and greater predicted specificity (off-target).

### Study participant

The patient was a 47-year-old female who presented with progressive bilateral hand tremor, manifesting as postural and voluntary action-induced tremor and mild myoclonus, which she had initially noticed in her early twenties. Her past medical history was unremarkable, with no history of generalized seizures. Electroencephalography revealed no abnormalities. Electrophysiological hallmarks that typically confirm the cortical origin of tremors, including giant somatosensory evoked potentials (SEPs), C-reflexes, and a preceding cortical spike on jerk-locked back-averaging (JLA), are well recognized. We first conducted an SEP examination; however, at that time the patient’s clinical symptoms had completely disappeared following clonazepam treatment. Likewise, SEPs showed no giant responses. Consequently, further electrophysiological evaluations, including C-reflexes and JLA, were not performed because the clinical symptoms had resolved and SEPs had normal findings. A positive family history suggestive of autosomal dominant inheritance was reported by the proband, including a parent and a sibling who had been clinically diagnosed based on typical symptoms. Additionally, several of her children reportedly exhibited similar characteristic tremors; however, none had undergone a medical evaluation. These familial findings collectively support an autosomal dominant mode of inheritance. Brain magnetic resonance imaging revealed no abnormalities. Following clonazepam treatment, the patient’s symptoms improved. Although these clinical features were suggestive of FAME, a definitive diagnosis could not be made; therefore, detailed genetic testing was crucial.

## Results

**Fig. 1:**
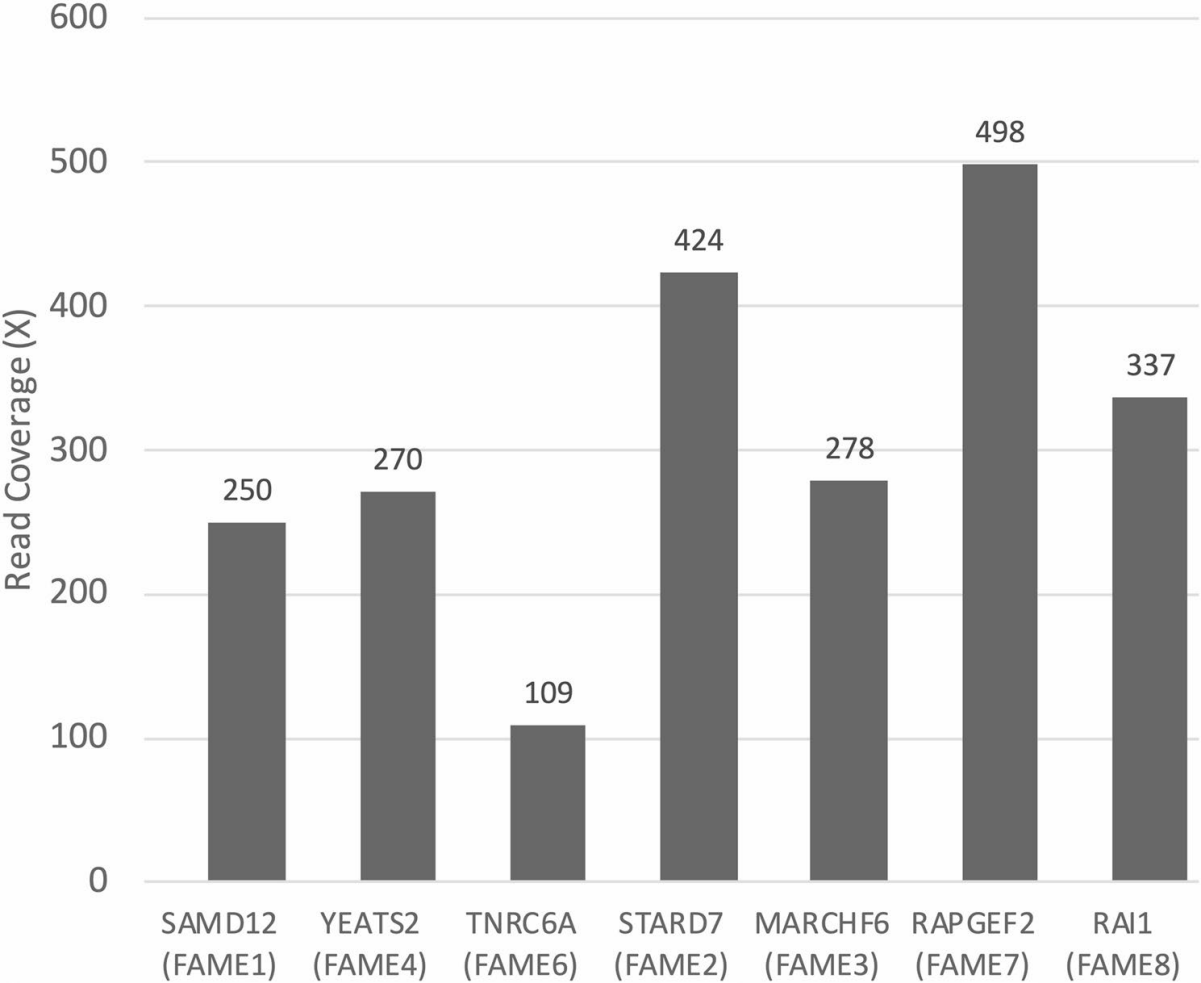
Read coverage of the target repeat loci. The x-axis indicates the target repeat loci, and the y-axis indicates read coverage at each locus

We developed a Cas9-mediated targeted long-read sequencing panel targeting seven known FAME-associated intronic repeat loci (*SAMD12*, *STARD7*, *MARCHF6*, *YEATS2*, *TNRC6A*, *RAPGEF2*, and *RAI1*) (Table 1). The corresponding guide RNAs were designed to flank each pentanucleotide repeat region and were pooled to form the FAME-associated repeat panel (Table 2). This panel successfully provided sufficient read coverage across all target regions and facilitated the accurate detection of repeat expansions (Fig. 1). We applied this panel to our patient (proband) who was clinically suspected of having FAME1 (BAFME) and presented with characteristic hand tremors beginning in her early twenties and had a strong family history.

**Table 2:** Guide RNA sequences, genomic coordinates, and target sizes in the FAME-associated repeat panel

Disease	gene	Plus strand (upstream)				Minus strand (downstream)					Target size (bp)
		crRNA sequence	Genome coordinate (hg38)	On-target score	Off-target score	crRNA sequence	Genome coordinate (hg38)	On-target score	Off-target score	target	
FAME1 (BAFME)	*SAMD12*	GTTTCGTTCTCTCCTTGAGC	chr8:118365468–118365487	56	52	TTTTGGATGAGTAACCTAAT	chr8:118368344–118368363	85	46	chr8:118366815–118366918	2895
FAME2	*STARD7*	AACCCAAAAGATAGCCACAC	chr2:96195312–96195331	71	42	GGCAATTGGAACCAAGGATG	chr2:96198530–96198549	81	20	chr2:96197066–96197124	3237
FAME3	*MARCHF6*	AATCCTTGACACCTATAAGC	chr5:10354510–10354529	76	62	GGGAAGACTTACTATACGCA	chr5:10356770–10356789	66	77	chr5:10356339–10356411	2279
FAME4	*YEATS2*	GGACAAGTTTAGGTATCTGC	chr3:183710951–183710970	85	69	TAGTATTAGCCACAATCCTA	chr3:183712738–183712757	88	58	chr3:183712187–183712226	1806
FAME6	*TNRC6A*	CCAAACGTCTGACTCTCTGT	chr16:24611510–24611529	87	68	TCACGGGATCTCAAGTCTAC	chr16:24615330–24615349	87	72	chr16:24613438–24613532	3839
FAME7	*RAPGEF2*	ATAAGAGTGATGACGTACTT	chr4:159341171–159341190	81	74	AAATTTGGGTTTAACACCTG	chr4:159344476–159344495	79	43	chr4:159342526–159342618	3324
FAME8	*RAI1*	GTAGGGTGTGGACGTGCAAA	chr17:17806504–17806523	70	69	ACGTCGTCTTGAGATTCCAG	chr17:17810343–17810362	75	83	chr17:17808359–17808460	3858

On-target and off-target scores were obtained from the IDT Alt-R CRISPR-Cas9 guide RNA design tool. The on-target score indicates the predicted cleavage efficiency at the intended locus, whereas the off-target score indicates the predicted specificity (on a 0–100 scale, with higher values indicating better performance)

Using the GridION platform with a single MinION flow cell, sequencing was performed. Cas9 enrichment yielded sufficient coverage across all targeted loci, with a mean (± standard deviation) coverage of 309.4 ± 126.3 and a median of 278 per locus (Fig. 1 and Supplementary File 1. Among the seven loci, the *SAMD12* region demonstrated particularly high enrichment efficiency, facilitating the precise resolution of the repeat structure.

Using LAST-TRAIN, per-base error parameters were estimated [15]. The substitution probability matrix and insertion/deletion existence and extension values are presented in Supplementary File 2. Diagonal values of the substitution matrix, indicating appropriate base calls, were consistently high, whereas off-diagonal values, denoting substitution errors, were extremely low (Supplementary File 1 and 3).

**Fig. 2:**
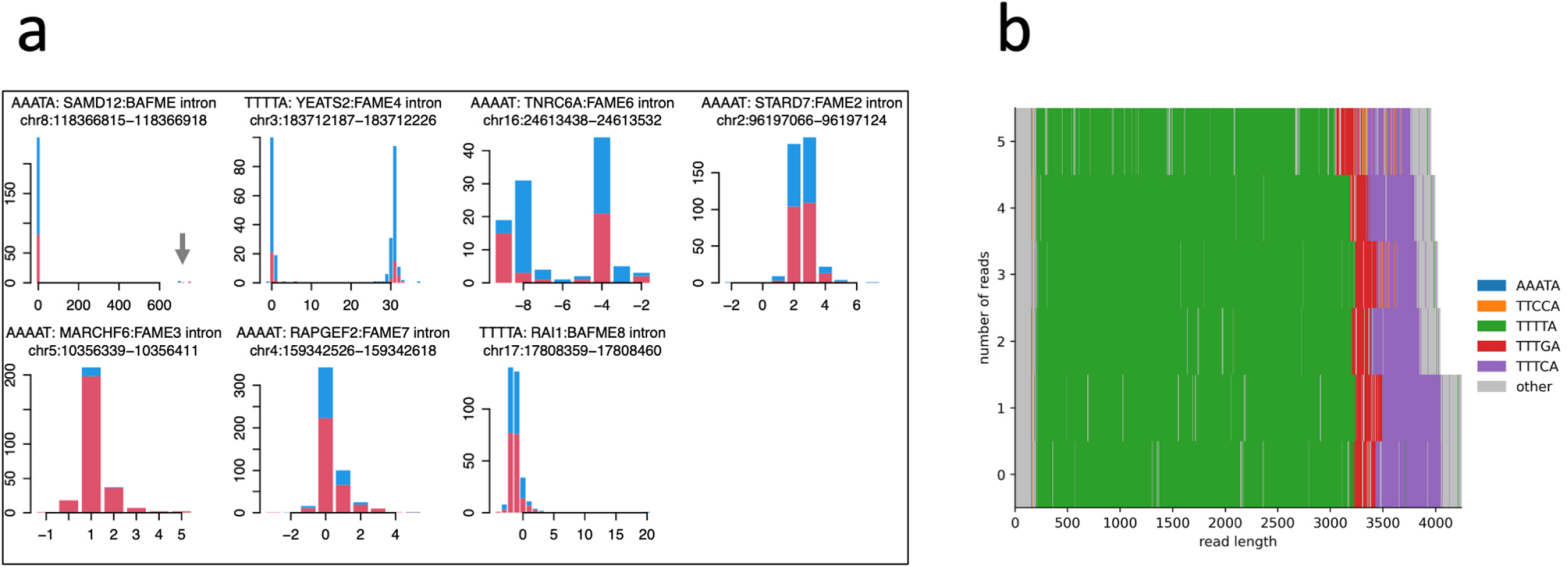
Detection and characterization of pathogenic repeat expansions in *SAMD12.* **a** Histograms showing changes in the copy number of the familial adult myoclonic epilepsy-associated pentanucleotide repeat loci. A pathogenic repeat expansion is observed at the *SAMD12* locus (arrow). The y-axis indicates read count, and the x-axis indicates changes in copy number relative to the reference genome (GRCh38). Reads mapped to the forward strand (red) and reverse strand (blue) are separately shown. **b** Internal repeat sequences of the expanded allele. TTTTA, TTTGA, and TTTCA repeat units are presented in green, red, and purple, respectively. The x- and y-axes indicate read length (base pairs) and the number of reads, respectively

Pathogenic intronic repeat expansion was identified at the *SAMD12* locus in the patient using the tandem-genotypes tool (Fig. 2a, b). The expanded allele contained several TTTCA/TTTTA motifs, consistent with previously reported pathogenic expansions in FAME1, and the TTTGA motif was also observed. The other six loci demonstrated no significant repeat expansions. Based on the crude allele prediction option (-o2) of tandem-genotypes, the expanded allele at the *SAMD12* locus comprised approximately 689 additional repeats relative to the reference genome.

## Discussion

CRISPR/Cas9-enriched long-read sequencing has been applied for identifying various conditions caused by repeat expansions, including spinocerebellar ataxia [14, 16, 17], Friedreich ataxia, fragile X-associated tremor/ataxia syndrome, and cerebellar ataxia, neuropathy, vestibular areflexia syndrome [17]. In this study, we developed a Cas9-mediated targeted long-read sequencing panel that can detect pathogenic pentanucleotide repeat expansions across all seven currently known FAME-associated genes. Our panel identified a pathogenic repeat expansion in *SAMD12* in a patient with clinical features of FAME. This approach provides a proof-of-concept molecular diagnostic tool, particularly for patients with suspected FAME in whom conventional sequencing techniques, including Sanger sequencing and short-read NGS, frequently experience difficulties in detecting causative expansions, especially when these are very long (e.g., over 1,000 base pairs), owing to their repetitive nature.

Flexibility and scalability represent the key advantages of this panel design. As new FAME-associated loci are discovered, additional guide RNAs can be readily incorporated into the panel, facilitating rapid adaptation to reflect the evolving genetic landscape of the disorder. Simultaneously analyzing multiple repeat loci is particularly useful in cases with atypical presentations or an unclear family history. Furthermore, this panel may enable earlier and more accurate diagnosis than conventional electrophysiological assessments, thereby guiding therapeutic strategies and enabling appropriate genetic counseling for affected families.

An important feature of the present case is that electrophysiological hallmarks typically used for confirming the cortical origin of tremors, including giant SEPs, C-reflexes, and JLA, were not demonstrated because the patient had already achieved complete symptom control with clonazepam upon evaluation. In routine clinical practice, such early treatment is necessary for alleviating symptoms, precluding the demonstration of electrophysiological findings required for a definitive diagnosis. Thus, the use of a comprehensive genetic panel offers a major clinical advantage. The targeted long-read sequencing panel developed in this study facilitated an unambiguous molecular diagnosis of FAME, even when electrophysiological criteria were not fulfilled, underscoring its practical utility in real-world clinical settings.

Coverage analysis revealed that enrichment efficiency varied between loci, with per-locus median coverage values summarized in Supplementary File 1. This finding indicates moderate variability consistent with the anticipated performance of Cas9-based enrichment. As shown in Fig. 2a, the expanded allele exhibits lower coverage than the nonexpanded allele. This observation aligns with previous findings by Mizuguchi et al. [18], who reported that repeat size was negatively correlated with the proportion of expanded allele reads. Similarly, in our experience, the number of reads supporting the expanded allele decreases as the repeat length increases. Although the underlying mechanism remains unelucidated, this suggests that sequencing bias can become more pronounced in cases with huge expansions, and expanded alleles can generate fewer high-quality reads. Such bias should be recognized as a potential limitation in future clinical applications. Systematic assessment across various patients will be required to characterize further between-sample variability, which we have noted as a limitation of the present study.

Our clinical findings being based on a single proband represents a major limitation of this study. Although FAME is a rare disorder, additional cases are necessitated to completely establish the robustness, sensitivity, and specificity of the panel. In this study, we successfully demonstrated the feasibility and diagnostic value of the targeted long-read sequencing approach. In the future, to further evaluate the sensitivity, specificity, and clinical utility of the panel in diverse FAME populations, we aim to apply this panel to other affected individuals within the same family and conduct broader screening in larger cohorts. These investigations may facilitate the integration of genetic testing into standardized diagnostic criteria for FAME.

Interestingly, the repeat expansion observed in this case comprised a rare combination of TTTTA, TTTGA, and TTTCA motifs. Although this combination has been previously reported [18], this finding emphasizes the structural diversity of repeat motifs in FAME1, highlighting the significance of employing sequencing methods capable of resolving complex repeats.

It is worth noting several technical considerations that may be relevant to future applications of this approach: First, in this study, genomic DNA was extracted from peripheral blood leukocytes using the QIAamp DNA Blood kit (Qiagen), followed by purification and concentration with AMPure XP magnetic beads (Beckman Coulter). This protocol yielded DNA of sufficient integrity for Cas9 enrichment and nanopore sequencing, demonstrated by the successful detection of the expanded *SAMD12* allele. Although we acknowledge that ultra-high molecular weight DNA extraction protocols (e.g., Nanobind or phenol–chloroform methods) may offer longer fragments and potentially enhance the yield of ultra-long reads, the present approach was sufficient for the targeted long-read sequencing panel applied in this study. Second, newer R10.4.1 flow cells may demonstrate varying performance characteristics, particularly in repetitive regions and should be evaluated in future studies. Finally, various basecaller versions may process long intronic repeats differently, representing a potential limitation for broader clinical applications.

Per-base error parameters estimated using LAST-TRAIN were within the expected range for Oxford Nanopore sequencing, confirming that the data quality of Cas9-enriched reads was sufficient for reliable downstream analysis and enabled an unambiguous molecular diagnosis in this case. Importantly, multiple long reads successfully spanned the expanded *SAMD12* allele with both flanking sequences, allowing direct visualization of the motif structure and approximate repeat size estimation. These results demonstrate that, despite the characteristic indel errors of nanopore sequencing, the pathogenic expansion can be reliably detected. Future improvements in sequencing chemistry and basecalling algorithms are expected to further reduce indel errors, and platforms with intrinsically lower error rates, such as PacBio HiFi, may also provide complementary advantages.

## Conclusions

Our targeted long-read sequencing panel facilitates the detection of intronic repeat expansions in patients with FAME1 (also referred to as BAFME). This panel offers a promising proof-of-concept molecular tool that, with further validation, may serve as a clinically valuable method for the molecular diagnosis of FAME1 and the genetic diagnosis of other FAME subtypes.

## Supplementary Information

Supplementary Material 1. Locus-specific coverage depth of the FAME repeat panel.

Supplementary Material 2. Per-base error profile estimated using LAST-TRAIN.

Supplementary Material 3. Representative IGV view of Cas9-enriched nanopore long-read alignments at the *SAMD12* locus.

## Data Availability

The sequence reads mapped to the target region and analyzed during the current study have been deposited in the DDBJ Sequence Read Archive (DRA), which is part of the International Nucleotide Sequence Database Collaboration (INSDC), under the BioProject accession number PRJDB35726.

## Abbreviations

FAME: Familial adult myoclonic epilepsy
BAFME: Benign adult-onset familial myoclonic epilepsy
NGS: Next-generation sequencing
SEPs: Somatosensory evoked potentials
JLA: Jerk-locked back-averaging
