# Supplementary material for: Targeted nanopore long-read sequencing panel for the molecular diagnosis of intronic expansion in familial adult myoclonic epilepsy
Source: BMC Med Genomics. 2025 Nov 11;18:180. doi: 10.1186/s12920-025-02247-9 (PMC12607150; doi:10.1186/s12920-025-02247-9)
Supplement: Supplementary file 1 — Supplementary Material 1. Locus-specific coverage depth of the FAME repeat panel. [file 12920_2025_2247_MOESM1_ESM.docx]

**Supplementary File 1. Locus-specific coverage depth of the FAME repeat panel**

| Gene  (FAME subtype) |  | Read coverage (×) |
| --- | --- | --- |
| *SAMD12* (FAME1) |  | 250 |
| *YEATS2* (FAME4) |  | 270 |
| *TNRC6A* (FAME6) |  | 109 |
| *STARD7* (FAME2) |  | 424 |
| *MARCHF6* (FAME3) |  | 278 |
| *RAPGEF2* (FAME7) |  | 498 |
| *RAI1* (FAME8) |  | 337 |
| Mean ± SD |  | 309.4 ± 126.3 |
| Median (IQR) |  | 278 (260–380) |

This table summarizes the per-locus median coverage values acquired from the targeted Cas9-enrichment long-read sequencing panel in the analyzed proband. Coverage ranges from 109× (*TNRC6A*/FAME6) to 498× (*RAPGEF2/*FAME7), with a mean of 309× (standard deviation [SD], 126), a median of 278×, and an interquartile range (IQR) of 260–380×. These values indicate moderate variability between loci, consistent with the anticipated performance of Cas9-based enrichment. See **Figure 1,** where these per-locus coverage values are graphically presented.
