# Supplementary material for: Targeted nanopore long-read sequencing panel for the molecular diagnosis of intronic expansion in familial adult myoclonic epilepsy
Source: BMC Med Genomics. 2025 Nov 11;18:180. doi: 10.1186/s12920-025-02247-9 (PMC12607150; doi:10.1186/s12920-025-02247-9)
Supplement: Supplementary file 2 — Supplementary Material 2. Per-base error profile estimated using LAST-TRAIN. [file 12920_2025_2247_MOESM2_ESM.docx]

**Supplementary File 2. Per-base error profile estimated using LAST-TRAIN**

|  | Read_A |  | Read_C |  | Read_G |  | Read_T |
| --- | --- | --- | --- | --- | --- | --- | --- |
| Ref_A | 0.29000 |  | 0.00067 |  | 0.00480 |  | 0.00042 |
| Ref_C | 0.00071 |  | 0.20000 |  | 0.00028 |  | 0.00130 |
| Ref_G | 0.00490 |  | 0.00028 |  | 0.20000 |  | 0.00033 |
| Ref_T | 0.00041 |  | 0.00130 |  | 0.00027 |  | 0.29000 |

Substitution probability matrix estimated using LAST-TRAIN from on-target reads. Each value represents the probability of a given reference base (rows) being called as a particular read base (columns). Diagonal values indicate correct base calls, while off-diagonal values indicate substitution errors. Probabilities of substitution, deletion, and insertion between human DNA reads and a reference human genome (hg38). Diagonal values denote correct base calls, whereas off-diagonal values represent substitution errors. Base deletion probability: existence = 0.017, extension = 0.49. Base insertion probability: existence = 0.013, extension = 0.50.
