# Supplementary material for: Targeted nanopore long-read sequencing panel for the molecular diagnosis of intronic expansion in familial adult myoclonic epilepsy
Source: BMC Med Genomics. 2025 Nov 11;18:180. doi: 10.1186/s12920-025-02247-9 (PMC12607150; doi:10.1186/s12920-025-02247-9)

**Supplementary File 3. Representative IGV view of Cas9-enriched nanopore long-read alignments at the *SAMD12* locus**

This IGV snapshot illustrates nanopore long-read alignments at the targeted *SAMD12* locus (chr8:118365468–118368363). Visualization was performed using Integrative Genomics Viewer (IGV, <https://igv.org/>). Each horizontal gray bar represents an individual read. Purple marks indicate small insertions, while gaps within the reads (shown as horizontal lines) indicate deletions. The top gray histogram shows coverage depth.


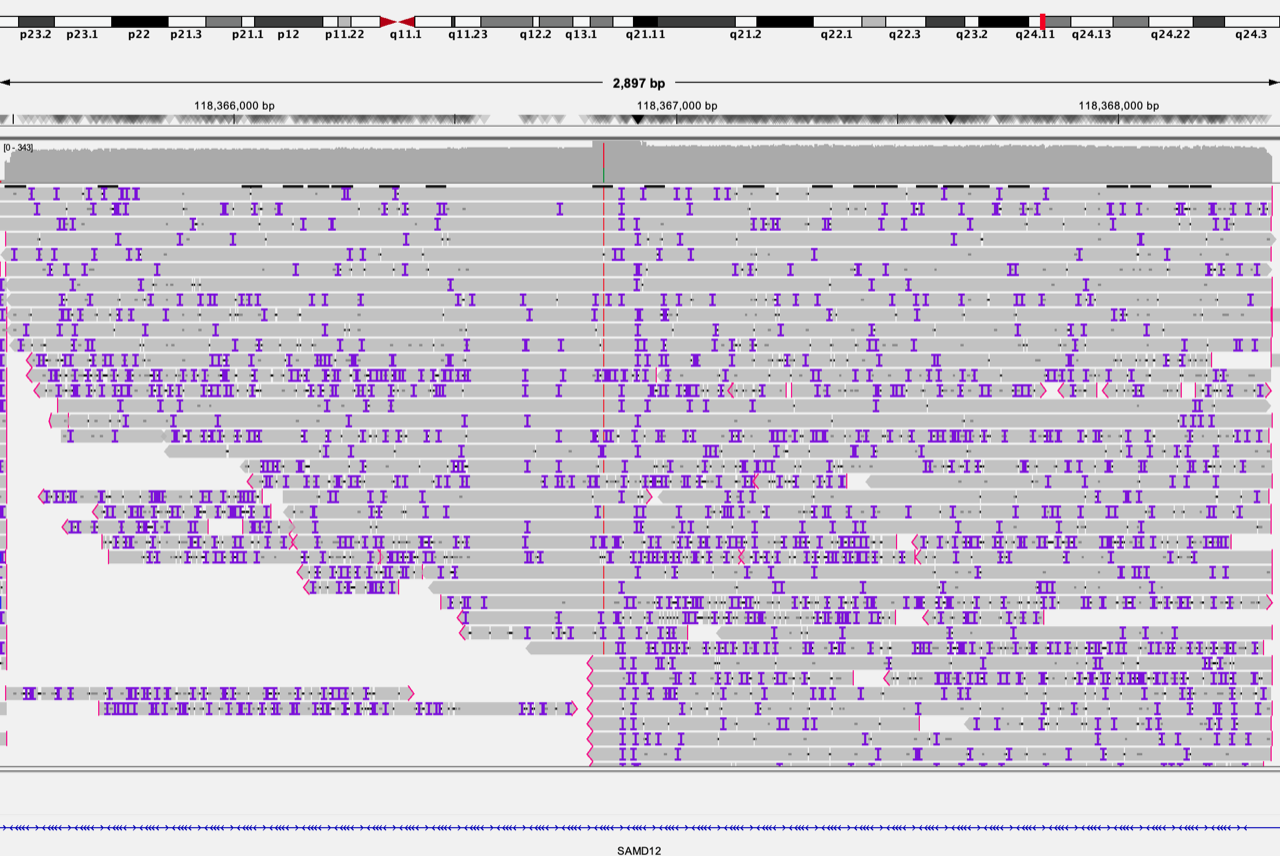

Supplement: Supplementary file 3 — Supplementary Material 3. Representative IGV view of Cas9-enriched nanopore long-read alignments at the SAMD12 locus. [file 12920_2025_2247_MOESM3_ESM.docx]
